# Could heart rate recovery and exercise capacity predict abnormal 99mTc-MIBI myocardial perfusion scan findings?

**DOI:** 10.21542/gcsp.2021.7

**Published:** 2021-04-30

**Authors:** Aryan Naghedi, Nasim Namiranian, Mohsen Goudarzi, Reza Nafisi Moghadam, Seid Kazem Razavi-Ratki

**Affiliations:** 1 Faculty of Medicine, Shahid Sadoughi University of Medical Sciences, Yazd, Iran; 2 Yazd Diabetes Research Center, Shahid Sadoughi University of Medical Sciences, Yazd, Iran; 3 Department of Radiology, Shahid Sadoughi Hospital, Faculty of Medicine, Shahid Sadoughi University of Medical Sciences, Yazd, Iran

## Abstract

**Background and aim**: Ischemic heart diseases lead to numerous deaths worldwide. Prevention, rapid diagnosis and treatment are principal strategies in controlling mortality due to coronary artery diseases (CAD). Considering variety of options, finding suitable diagnostic modality for each patient has been controversial for long times. Exercise treadmill test (ETT) is a well-known and old modality compared to radionuclide scintigraphy. In this study we aimed to assess if heart rate recovery (HRR) and exercise capacity (EC) in ETT can predict perfusion defects in 99mTc-MIBI SPECT myocardial perfusion imaging (MPI).

**Materials and methods**: In this cross-sectional, descriptive-analytic study, we enrolled 254 patients referred for MPI to nuclear medicine department of Afshar or Shahid Sadoughi Hospital, Yazd, Iran. All patients underwent ETT and MPI. HRR and EC in ETT plus abnormal perfusion findings in MPI along with patient’s history and demographic information were recorded in a questionnaire. Finally, all data were analyzed using SPSS ver.22 software.

**Results**: Based on our results, 161 (63.4%) of patients were men. Half of patients were diagnosed with hypertension. 45% were diagnosed with diabetes, 41% had hyperlipidemia and 8.3% were smokers. Based on our findings, 18.1% of patients had abnormal MPI results. Our analytic results indicated that there is no statistically significant association between transient RV visualization, transient ischemic dilation or lung to heart ratio with 1^st^ and 2^nd^ minute HRR and EC.

**Conclusion**: Considering lack of association between ETT indices and MPI findings, it seems that ETT is not adequately sensitive in predicting perfusion defects in MPI. Thus, it seems logical in some cases to ignore expenses of MPI and suggest it to patients before ETT based on clinical judgement.

## Introduction

Ischemic heart disease is the most common causes of mortality and morbidity worldwide^[1]^. Myocardial ischemia is defined as impaired perfusion to myocardium. This condition happens during imbalance between oxygen demand and supply. The most common etiology for ischemia is epicardial coronary artery atherosclerosis^[2–4]^.

The most important strategy to control coronary artery incidence and its related mortality is to detect and control risk factors or initial disease rapidly^[5]^. Nowadays a lot of diagnostic modalities are being used for achieving this goal such as imaging, laboratory tests and stress tests. Based on recent literature high risk patients for cardiovascular diseases undergo more than one diagnostic test^[6]^.

Although still clinical and physical examination is the principal diagnostic tool for diagnosis of coronary artery disease, it is sometimes challenging for physicians to make a certain diagnostic or treatment strategy in some susceptible cases. This is where clinical entanglement begins to pick the best diagnostic test. Among these tests, exercise treadmill test (ETT) and radionuclide scintigraphy tests are mentionable^[7–9]^.

ETT is a well-known test which is spreadingly being used for long time for detecting coronary artery disease in high risk patients. By help of this test, clinically applicable evidences of ischemia can be extracted from patients with susceptible coronary artery disease. In literature it is said that ETT with a sensitivity of 68% and specificity of 77% can help physicians for this goal. Among different items in ETT, heart rate recovery (HRR) and exercise capacity (EC) are being discussed recently. Their important in diagnosis and prognosis of patients is still controversial among researchers^[10,11]^.

Radionuclide scintigraphy is a newer and more expensive test compared to ETT but provides a higher sensitivity and specificity. Images are mostly obtained by Thallium 201 injection in maximum exercise activity. Other radionuclide agents are also being used such as 99mTc-MIBI. This imaging modality is not that well known as ETT and its accurate clinical benefits are not explained. In other words, the exact position of this test is not an agreement point among clinicians^[12,13]^.

Based on all mentioned above, after reviewing literature, we found little evidences comparing ETT and radionuclide scintigraphy modalities in diagnosis of coronary artery disease (CAD) in susceptible patients. Thus, here we aimed to investigate possible association between heart rate recovery and exercise capacity in ETT with abnormal 99mTc-MIBI SPECT/MPI myocardial perfusion scan findings.

## Material and Methods

This study is a cross-sectional, descriptive-analytic study performed on 254 consecutive patients referred to nuclear medicine department of Afshar or Shahid Sadoughi hospital, Yazd, Iran for myocardial perfusion scan. An informed consent was obtained from all enrolled patients and the study was designed and performed based on latest declaration of Helsinki. This study is also registered in research ethics committee of Shahid Sadoughi University of Medical Sciences, Yazd, Iran and is approved with IR.SSU.MEDICINE.REC.1397.017 approval ID.

We included all susceptible patients for CAD undergoing myocardial perfusion imaging (MPI). Patients with contraindication for ETT, history of percutaneous coronary intervention or coronary artery bypass grafting were excluded. we also excluded patients consuming agents interfering with heart rate such as β-blockers or those who were not able to finish ETT.

All patients underwent ETT with standard protocol. After achieving 85% of defined heart rate 99mTc-MIBI was injected and stress test continued for 1 more minute. Finally, patients’ HRR was recorded 1 minute and 2 minutes after finishing ETT and then, patients underwent MPI via SPECT device with Cedar Sinai software.

Patients demographic and history along with ETT and MPI results were recorded in a questionnaire. All patients’ data entered SPSS ver.22 software and data were analyzed using independent sample T test. In all tests a *P*-value < 0.05 was considered to be statistically significant.

## Results

Results of this study showed that 161 (63.4%) patients were women and 93 (36.6%) of them were men. In this study population 127 (50%) patients had hypertension (HTN), 114 (45%) patients had diabetes mellitus (DM), 21 (8.3%) were smokers and 105 (41%) patients had hyperlipidemia (HLP). 23 (9.1%) of patients mentioned a history of myocardial infarction.

Our ETT indices such as 1^st^ minute HRR, 2^nd^ minute HRR and EC in METs score are reported as minimum, maximum and mean ±SD in Table 1.

**Table 1:** Descriptive results related to ETT findings in study population.

	Minimum	Maximum	Mean ±SD
1^st^ minute HRR	3	80	34 ±13
2^nd^ minute HRR	1	94	42 ±15
EC (METs)	1	17	10.9 ±2.3

MPI results are reported in Table 2. A five-point scale was used for scoring the results. Prevalence of transient right ventricular (RV) visualization, transient ischemic dilation (TID) and lung to heart ratio (LHR) are also reported in this table.

**Table 2:** Descriptive statistic table related to different abnormal MPI findings. (0: normal, 1: mild ischemia, 2: moderate ischemia, 3: severe ischemia, 4: no perfusion).

Perfusion defect	Frequency	Percentage
0	208	81.9%
1	15	5.9%
2	6	2.4%
3	13	5.1%
4	12	4.7%
Total	254	100%
Transient RV visualization	3	1.2%
TID	9	3.5%
LHR	2	0.8%

Analytic results based on association between ETT indices and transient RV visualization showed that there is no statistically significant association between transient RV visualization and ETT markers (1^st^ minute HRR *P*-value = 0.7, 2^nd^ minute HRR *P*-value = 0.6 and EC *P*-value = 0.08). Table 3 indicates results of transient RV visualization.

**Table 3:** Association between ETT indices and transient RV visualization.

	Transient RV visualization	Mean ±SD	*P*-value
1^st^ minute HRR	YES	26 ±17.3	0.7
	NO	34 ±13.8	
2^nd^ minute HRR	YES	24 ±23	0.6
	NO	42 ±15.5	
EC (METs)	YES	5.7 ±2.4	0.08
	NO	11.04 ±2.2	

We also searched for any possible association between ETT markers and TID in MPI. Again our results showed lack of statistical correlation between these two (1^st^ minute HRR *P*-value = 0.4, 2nd minute HRR *P*-value = 0.5 and EC *P*-value = 0.9). The results are summarized in Table 4.

**Table 4:** Association between ETT indices and TID.

	TID	Mean ±SD	*P*-value
1^st^ minute HRR	YES	36.2 ±11.45	0.4
	NO	34.3 ±14	
2^nd^ minute HRR	YES	35 ±13.2	0.5
	NO	42.5 ±15.7	
EC (METs)	YES	10 ±2	0.9
	NO	11 ±2.3	

At the end we assessed association between LHR in MPI and same ETT markers mentioned above. Still we couldn’t conclude any relationship between ETT indices and LHR (1st minute *P*-value = 0.66, 2nd minute *P*-value = 0.23 and EC *P*-value = 0.09). Detailed results are reported in Table 5.

**Table 5:** Association between ETT indices and LHR.

	LHR	Mean ±SD	*P*-value
1^st^ minute HRR	YES	23.5 ±12	0.66
	NO	34.5 ±13.9	
2^nd^ minute HRR	YES	30 ±7	0.23
	NO	42.3 ±15.7	
EC (METs)	YES	8.85 ±0.7	0.09
	NO	10.9 ±2.3	

## Discussion

There are accurate and non-invasive tests for evaluating severity of coronary artery disease in susceptible patients. By means of these modalities, clinicians can avoid unnecessary invasive modalities such as coronary angiography. In this field, ETT is considered in mind among first choices since long ago. Considering non-invasive entity of nuclear myocardial perfusion scan same as ETT, in this study we aimed to investigate any possible association between these two.

In this study we did not find any statistically significant association between studied indices of ETT (1^st^ minute HRR, 2^nd^ minute HRR and EC) and abnormal MPI findings in susceptible patients for coronary artery disease.

MPI is a new modality compared to ETT and is performed by injecting a safe radionuclide agent. Myocardial perfusion scan is assessed in both resting and exercise phases. Preparations for SPECT MPI are the same as ETT including physical readiness and holding consumption of some medications for a short period^[14–16]^.

In a study performed by Bokhari et al. on patients with angina pectoris, they concluded that MPI possess a higher sensitivity and specificity compared to ETT and they suggested that this modality can be used as a first line method to obtain more reliable results about condition of coronary arteries^[17]^.

Our results confirm Bokhari et al. findings. Although HRR and EC are powerful and independent predictors for cardiovascular death, as even explained in our previous studies, but they cannot be replaced with MPI.

In a study performed by Pedersen et al. which was published on 1991 in European heart journal, the researchers screened 2500 asymptomatic patients with ETT. Abnormal ETT was seen in 55 of them and only 9 patients (16%) were involved with cardiovascular disease. They concluded that ETT is not a good diagnostic test for cardiovascular diseases and cannot be used solely for this aim. In our study we included patients referred for MPI with abnormalities of ECG during ETT and those patients had mostly other risk factors too; and this means that our study sample consisted of patients with moderate risk of cardiovascular diseases^[18]^.

Sabharwal et al. performed a very interesting study in 2007 comparing ETT and SPECT/MPI. They tried to perform a cost analysis study in a randomized trial manner. They carried out this study on 457 patients with stable chest pain and susceptible for coronary artery disease. They found that mean cost for ETT was £490.44 and this amount was £512.41 for MPI. They concluded that there was no significant cost difference between initial ETT and MPI in intermediate or high likelihood patients ^[19]^.

As we have searched over online databases and based on our knowledge, there are not much reliable studies performed in this field and it seems that MPI and overally nuclear imaging is not at the center of attention as older modalities. For the same reason number of patients undergoing MPI are not a lot even in referral medical centers as ours.

We strongly suggest further researchers to perform similar studies with a higher study population. Studies which can compare MPI and ETT with gold standard modality (angiography) will be really helpful.

## Conclusion

We designed and performed a study on association between ETT indices and abnormal MPI findings in a sample size of 254 patients. Based on our knowledge there are not much studies performed in field of cardiac nuclear imaging similar to ours.

Our results indicate that 1^st^ minute HRR, 2^nd^ minute HRR and EC in ETT are not associated with different perfusion defects detected via ^9^^9^Tc MIBI SPECT MPI. Considering similar preparations and similar safety of both modalities, it seems that despite higher expenses of MPI, it can be logical to suggest nuclear imaging to patients before ETT in some cases based on physician’s clinical judgement and MPI modality should be more in consideration by clinicians.
